# Herpes Simplex Virus-1 Fine-Tunes Host’s Autophagic Response to Infection: A Comprehensive Analysis in Productive Infection Models

**DOI:** 10.1371/journal.pone.0124646

**Published:** 2015-04-20

**Authors:** Abraam M. Yakoub, Deepak Shukla

**Affiliations:** 1 Department of Microbiology and Immunology, University of Illinois, Chicago, IL United States of America, 60612; 2 Department of Ophthalmology and Visual Sciences, University of Illinois Medical Center, Chicago, IL United States of America, 60612; UC Irvine Medical Center, UNITED STATES

## Abstract

Herpes simplex virus-1 (HSV-1) infection causes severe conditions, with serious complications, including corneal blindness from uncontrolled ocular infections. An important cellular defense mechanism against HSV-1 infection is autophagy. The autophagic response of the host cell was suggested to be regulated by HSV-1. In this study, we performed a detailed analysis of autophagy in multiple HSV-1-targeted cell types, and under various infection conditions that recapitulate a productive infection model. We found that autophagy was slightly inhibited in one cell type, while in other cell types autophagy maintained its basal levels mostly unchanged during productive infection. This study refines the concept of HSV-1-mediated autophagy regulation to imply either inhibition, or prevention of activation, of the innate immune pathway.

## Introduction

Herpes simplex virus-1 is a double stranded DNA virus associated with chronic infections in humans [1, 2]. HSV-1 infections may result in serious morbid, potentially mortal, conditions. HSV-1 corneal infections may cause herpes stromal keratitis (HSK), a condition that may advance to corneal opacity and blindness [1–3]. In fact, HSV-1 represents the leading infectious cause of corneal blindness worldwide [3, 4]. HSV-1 utilizes various pathways and strategies for entry into host tissues, including the cornea [3, 4], from which it may disseminate into the nervous system causing a lethal condition, herpes encephalitis [1–5]. Additionally, HSV-1 may cause severe neonatal infections [1, 5].

Macroautophagy (hereafter referred to as autophagy) is a catabolic cellular process, aimed at degradative removal of certain cytoplasmic components (proteins, or organelles) of the cell, or intracellular pathogens [6–10]. The autophagy pathway involves sequestration of a part of the cytosol inside isolation membranes. These membranes give rise to autophagosomes (double-membrane vesicles compartments), which then fuse with the lysosomes for cargo destruction [6–10]. Numerous stimuli trigger autophagy, such as starvation, hypoxia, hormones, certain drugs, and some infections [8–10].

In HSV-1 infection, induced autophagy responses were shown to lower the virulence of the virus in vivo [11, 12], and limit its replication in vitro [12, 13]. The ability of autophagy to degrade HSV-1 particles was also suggested [13]. Such findings stress the importance of understanding how the virus may regulate the autophagic response of the host to infection. It was previously suggested that the HSV-1 neurovirulence gene product ICP34.5 suppresses autophagy levels in the host by binding to the autophagy protein beclin1 [11, 13, 14]. Other studies, however, reported that HSV-1 infection can also induce autophagy [15, 16]. However such a response was only reported at high multiplicities of infection (MOIs) [15, 16]. Accurate autophagy analysis and interpretation are complicated by numerous factors, such as assay limitations [17–19], cell type specific effects [20, 21], and infection conditions including virus strains, multiplicities of infection (MOIs), and time point post-infection [11, 13–16]. These factors collectively urged us to further elucidate HSV-1-mediated regulation of autophagy.

In this study, we determined HSV-1-regulated autophagy levels of the host cells under various productive infection conditions. Interestingly, we found that autophagy may be inhibited or may remain unchanged significantly in most cell types tested. These results help attain a comprehensive, more accurate understanding of virus-mediated regulation of autophagy.

## Results

### Study Conception and Design

Study of autophagy in HSV-1 infection may have been hampered by factors as methodological limitations, reliance on single point evaluations, testing a particular cell type, or utilizing specific infection conditions (including virus strains, and MOIs) especially those that do not reflect a productive infection situation. Thus, in order to eliminate effects or phenomena that might be specifically associated with a particular cell type or infection condition, this study takes a comprehensive approach to address how HSV-1 infection regulates autophagy. We have studied HSV-1 infection in multiple cell types infectable by HSV-1 (e.g. epithelial, neuronal, and fibroblast cells) to address any possible cell type-specific effects. We have also used various conditions (multiple MOIs and durations of infection, and two viral strains) in the context of a productive infection model. Furthermore, to accurately determine autophagic flux we adopted many approaches to assess autophagic activity of the cells, in order to prevent errors or nonspecific effects that might be associated with an individual method, carefully considering the precautions and established guidelines to correctly interpret autophagic flux monitoring data and assay results [17–19, 22].

### Analysis of Autophagy in HSV-1 Infection of HeLa Cells

**Fig 1:**
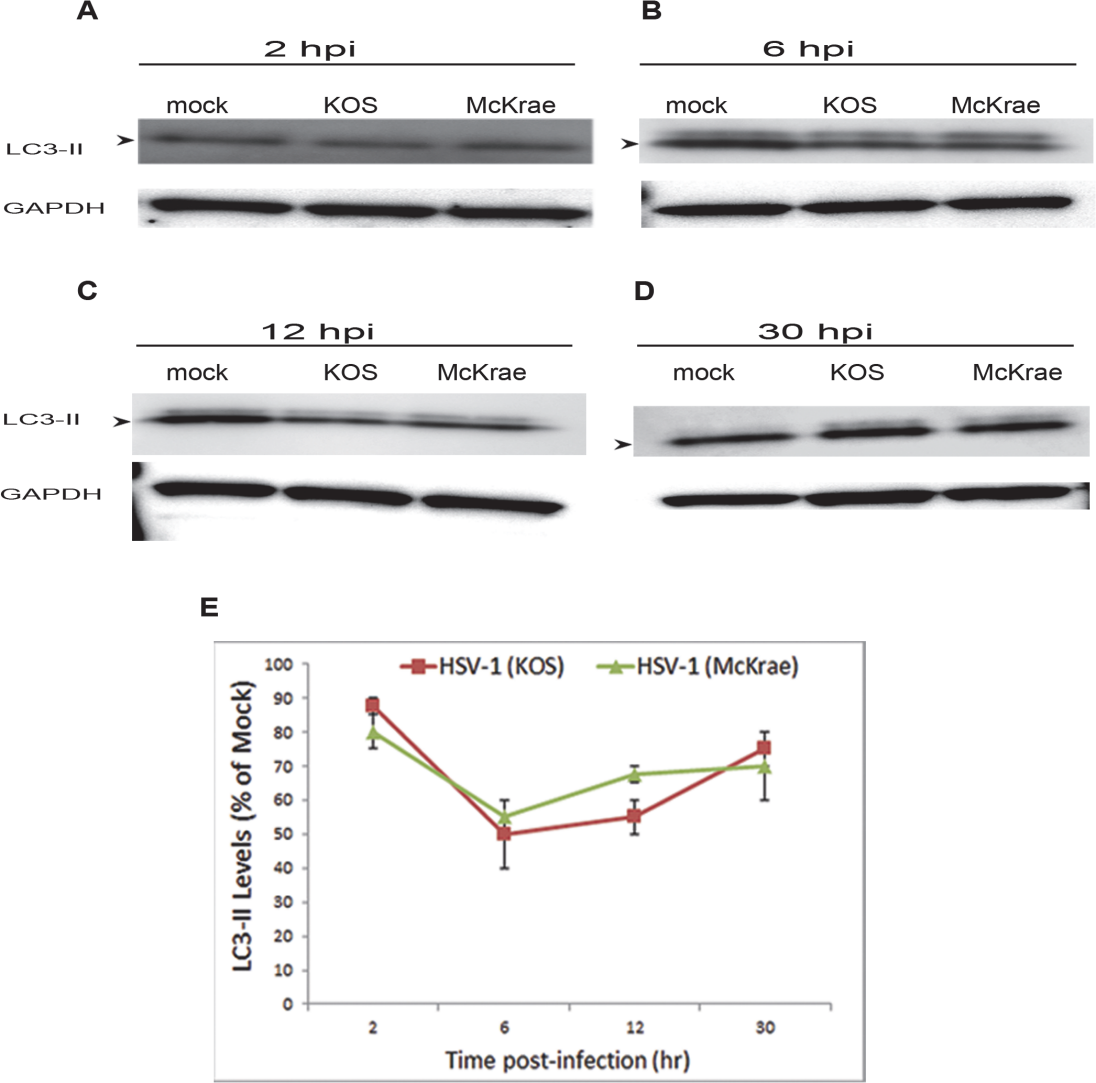
Analysis of Autophagy in HSV-1 Infection of HeLa Cells. A. HeLa cells were uninfected (mock), or infected with different strains of HSV-1 (KOS or McKrae). At 2 hpi, the cells were harvested, lysed, and the lysate was immunoblotted for LC3 levels. B. Uninfected or infected HeLa cells were harvested at 6 hpi, lysed, and immunoblotted. C. Immunoblotting of Uninfected or infected HeLa cells at 12 hpi. D. Immunoblotting of Uninfected or infected HeLa cells at 30 hpi. E. Quantification of LC3-II levels in (A-D), normalized to the housekeeping protein GAPDH.

To understand the regulation of autophagy in infection, we assessed the autophagic flux of the cells as a function of infection over time. Therefore we infected HeLa cells with the HSV-1 strains KOS or McKrae at MOI of 5. Mock- or HSV-1-infected cells were monitored at various time points after infection, to assess early and late responses. At each time point, autophagy was monitored via immunoblotting of microtubule-associated protein 1A/1B-light chain 3 (LC3)-II, an indicator of autophagic activity of the cells [23]. We found that autophagy levels were minimally inhibited at early points of infection (2 hours post-infection (hpi)) by both virus strains (Fig 1A and 1E). However, most effect (which did not exceed ~50% inhibition of the basal autophagy levels) was seen at 6 hpi (Fig 1B and 1E). At 12 and 30 hpi, gradual, and relative de-repression of autophagy was observed (Fig 1C, 1D and 1E). These results indicate that HSV-1 regulates autophagy in HeLa cells, via modest transient inhibition, which is consistent with previously reported observations in other systems [11, 14].

### Analysis of Autophagy in HSV-1 Infection of Fibroblast Cells

**Fig 2:**
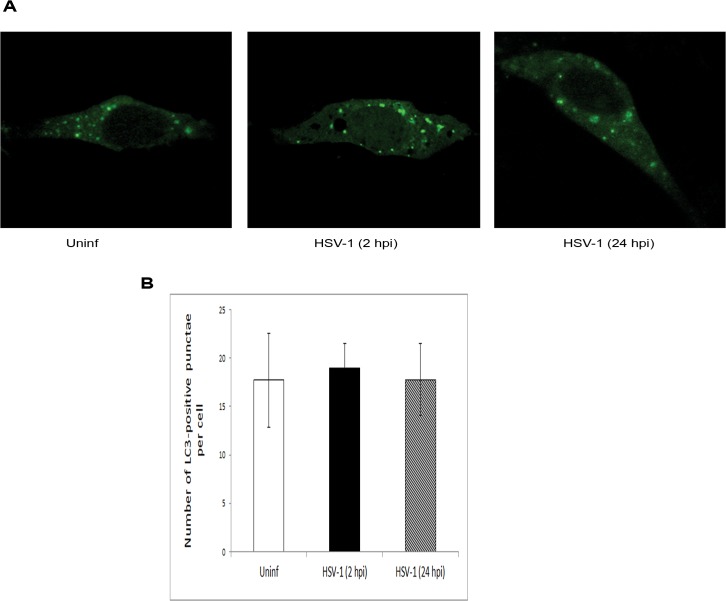
Analysis of Autophagy in HSV-1 Infection of Fibroblast Cells. A. MEFs were transfected with LC3-GFP. After 24 hr, the cells were uninfected or infected with different HSV-1. At 2 or 24 hpi, the cells were imaged using confocal microscopy. B. Quantification of the number of LC3-GFP punctae per cell in (A); about 30 cells per sample were counted.

Previous studies of autophagy in HSV-1 mostly used high MOIs for infection. Here, in order to more closely recapitulate a productive infection model, we used lower MOIs, such as 0.1 and 1. In order to microscopically monitor autophagy in mouse embryonic fibroblasts (MEFs), we transfected the cells with enhanced green fluorescent protein (EGFP)-LC3 plasmid. The cells were then infected with HSV-1 (KOS) and autophagy was assessed microscopically at early (2 hpi) and later (24 hpi) points of infection. We found that the cells at both time points did not show any significant change of autophagy levels upon infection, as manifested through observing the number and size of GFP-LC3 punctae (autophagosomes) per cell (Fig 2A and 2B).

### Analysis of Autophagy in HSV-1 Infection of Retinal Ganglion Cells

**Fig 3:**
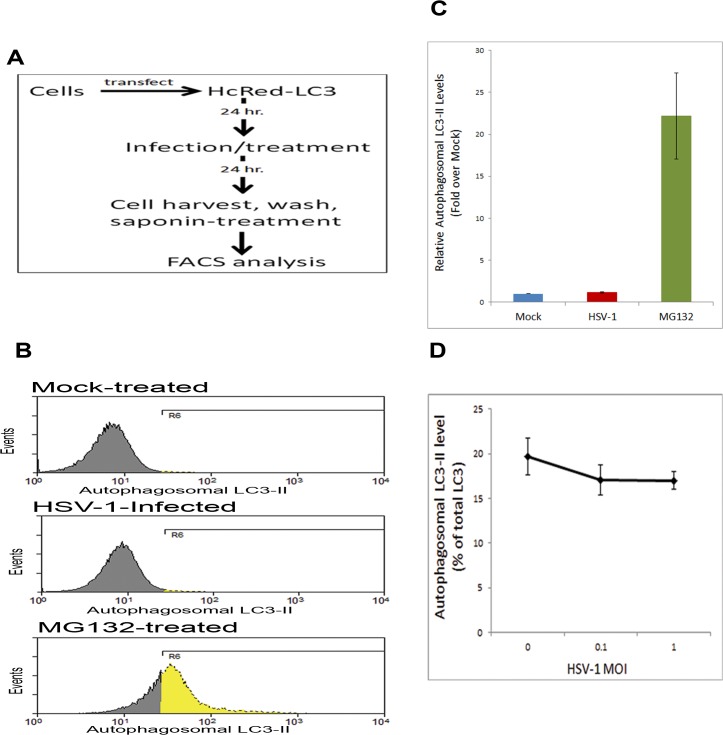
Analysis of Autophagy in HSV-1 Infection of Retinal Ganglion Cells. A. Experimental layout. B. RGC5 cells were transfected with HcRed-LC3. After 24 hr, the cells were mock-treated, treated with MG132, or infected with HSV-1 for 24 hr. The cells were washed in presence of saponin, and analyzed cytofluorimetrically. C. Quantitation of the relative levels of autophagosomal LC3-II in (B), calculated using integrated MFI of the gated region R6. D. Quantification of autophagosomal LC3-II (from saponin-treated cells), shown as % of total LC3 (non saponin-treated cells), as a function of infection with various MOIs of HSV-1 in RGC5 cells.

Using a retinal ganglion cell-like cell line (RGC5), we sought to determine the effect of HSV-1 infection on autophagy in neuronal cells. We also employed a novel, quantitative FACS-based assay of autophagy [24]. As outlined in Fig 3A, RGC5 cells were transfected with fluorescent LC3 plasmid. The cells were then mock-treated, infected with HSV-1, or treated with a known autophagy-inducing chemical MG132 [25, 26] as a positive control for autophagy activation. At 24 hpi, the cells were washed and then briefly treated with 0.05% saponin which solubilizes the non-autophagosomal LC3-I but does not affect the autophagosomal lipidated LC3-II protein (conjugated with phosphatidylethanolamine, becoming more hydrophobic and saponin-insoluble) [24]. In addition, saponin-untreated cells were used to quantify total LC3 levels in the cell, whereas the remaining fluorescence of the saponin-treated cells reflected the level of autophagosomal LC3-II [24]. Flow cytometry was then performed to determine autophagosomal LC3 levels in the cells. We did not find significant differences in autophagy flux between uninfected or infected cells, while MG132-treated cells showed remarkable induction of autophagy (Fig 3B and 3C). An MOI-response study showed that autophagy is affected only minimally at the different MOIs tested (Fig 3D).

### Analysis of Autophagy in HSV-1 Infection of Corneal Epithelial Cells

**Fig 4:**
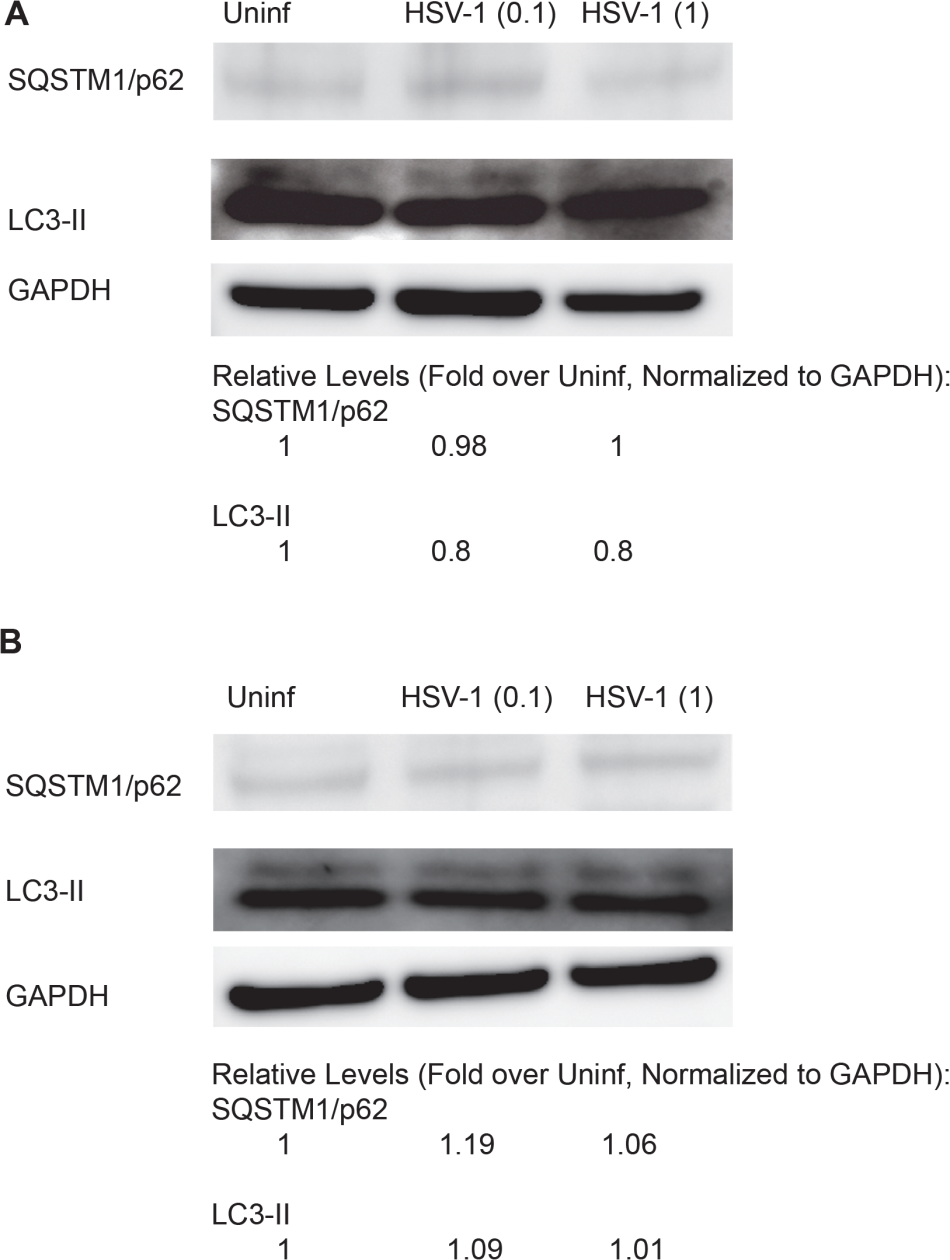
Analysis of Autophagy in HSV-1 Infection of Corneal Epithelial Cells. A. HCE cells were uninfected or infected with HSV-1 at the indicated MOIs. SQSTM1/p62 and LC3 were immunoblotted at 2 hpi. Quantification of band intensities was performed using digital band quantification software (ImageQunatTL; GE). B. Uninfected or infected HCE cells were immunoblotted at 8 hpi.

We then measured the effect of HSV-1 infection on autophagy in human corneal epithelial (HCE) cells, an important target of HSV-1 infection in vivo. The cells were infected with HSV-1 (KOS) at MOI of 0.1 or 1, and autophagy was monitored after 2 or 8 hpi. To assess autophagy, we measured, via immunoblotting, levels of LC3 and sequestosome1 (SQSTM1)/p62. Upon activation of the pathway, LC3-II levels increase while p62 is depleted due to its degradation by autophagy [17–19]. At all conditions, no significant changes in autophagy flux were observed, as monitored via LC3-II and p62 levels (Fig 4A and 4B).

## Discussion

Together, these various assays and conditions of infection in different cell types confirmed that HSV-1 infection may sometimes lower autophagy levels in the host; however autophagic activity of the host was in many cases maintained unchanged. Based on the results of this study, we propose a novel model to explain the regulation of autophagy in infection, whereby regulation of autophagy in productive HSV-1 infection may comprise inhibition or stabilization (maintaining steady state or near-steady state levels) of the pathway. This regulation implies prevention of autophagy activation, as inducing autophagy may result in viral clearance and reduction of infection [9–13]. Stimulating autophagy or enabling its activation (de-repressing it) in infection suppresses many viral infections and has recently been proposed as a novel mechanism for antiviral therapy [27, 28]. Thus, it is reasonable to propose that virus-mediated autophagy regulation or ‘fine-tuning’ implies either inhibition, or prevention of activation, of the pathway. Autophagy was shown to play proviral or antiviral roles in various viral infections. For example, in some DNA and RNA virus infections, induced autophagy directly enhanced virus replication, as is the case with influenza A and Dengue viruses [29, 30], or probably indirectly bolstered viral yields via suppressing apoptosis and death of infected cells, as is the case with human parvovirus B19 [31]. Conversely, the ability of autophagy to suppress some viral infections, directly via mediating viral degradation or indirectly via enhancing antigen presentation and adaptive immune responses, was also reported [9, 10, 13, 14]. Thus, fine-tuning of autophagy in HSV-1 infection might be a virus-attempted balance between the ‘bipartisan’ (proviral, and antiviral) functions of autophagy; however further work is required to investigate such a hypothesis.

In the productive infection systems tested, a significant fraction of the cell’s basal autophagy activity still persisted after infection (which fraction ranged from nearly 50–90% of basal levels, depending on the cell type and time point after infection). Thus far, it has not been clearly established whether this basal-level autophagy plays a role in HSV-1 infection, and the distinction between basal-level versus induced autophagy remains poor [6, 9, 32]. Learning from other systems and viruses [29–34], some hypotheses for the possible role of the fraction of autophagy remaining in infection may be speculated and are to be tested for HSV-1 infection by future studies. Differences in basal-level autophagy among cell types were reported, and importantly these basal autophagy differences were suggested to influence infection and virus yields in cells [35]. For example, enhanced basal autophagy in a murine cell line was associated with partial resistance of these cells to infection and lower viral yields [35]. It is thus reasonable to speculate that the virus regulates autophagy via inhibition or stabilization of autophagy, depending on the inherent constitutive autophagy levels in a cell type. Consistent with this hypothesis, HeLa cells reported to have high basal-level autophagy [19] showed autophagy inhibition (at least partially) upon infection (Fig 1). This, however, remains an important hypothesis yet to be investigated.

Previously, some work suggested that HSV-1 inhibits autophagy in certain cell types [11, 14]. To attain a more proper understanding of the HSV-1-autophagy relationship, the following facts ought to be considered. First, while it was reported that the viral factor ICP34.5 blocks autophagy [11, 14], another study suggested that ICP34.5 enhances viral yields via blocking translational arrest in infection, not via blocking autophagy [36]. Additionally, ICP34.5-independent autophagy was reported [37]. This ICP34.5-independent autophagy was dependent on presence of viral DNA, but not necessarily viral gene expression, in the cells [37]. However, such an autophagic response not antagonized by ICP34.5 was found in some non-permissive cell types [37], suggesting that the ICP34.5-mediated inhibition of autophagy varies with cell type. Second, HSV-1 (strain 17) mutants lacking the ICP34.5 beclin1-binding domain (this domain shown to inhibit autophagy) showed similar replication levels to wild-type virus in a neuroblastoma cell line [11], but less replication levels in vivo only at later points of infection [11]. These findings, along with the finding that deletion of the ICP34.5 beclin1-binding domain per se does not affect viral yields in vitro [36], suggest that ICP34.5 may play an autophagy-independent role that prevents viral clearance in late infection in vivo but does not affect early infection, such as control of adaptive immune responses. Third, significant variations in the ICP34.5 gene and protein sequences among commonly used laboratory strains were reported [38–41], and thus whether these inter-strain differences affect phenotypes observed should be evaluated. It is important to note that most ICP34.5-autophagy studies thus far involved the use of a particular HSV-1 strain (strain 17), known to differ from other strains in the ICP34.5 gene and protein sequences [38–41]. Fourth, a great number of HSV-1-autophagy studies utilized high MOIs for infection before assessing autophagic responses in the cell [15, 16]. These high MOIs (most of which do not truly recapitulate a productive infection model) and the extended time points (at which health of infected cells may be questionable) make it difficult to accurately segregate primary from secondary effects in infected cells and purely define autophagy effects [42]. Finally, the distinction between inhibition of autophagy (i.e. downregulating constitutive or basal autophagy) and inhibition of its stimulation in response to infection should be clearly drawn.

In conclusion, this study determines the effect of HSV-1 infection on autophagy flux in various cell types under productive infection conditions. While autophagy was modestly inhibited by the virus in some cell types and at certain points of infection, it remained unchanged significantly in other cell types and at most points of infection. This work suggests the importance of ‘fine-tuning’ autophagy to regulate viral infections, which may enhance our understanding of the complex autophagic regulation of herpesvirus pathogenesis.

## Materials and Methods

### Cells and cell culture

HeLa cells were obtained from American Type Culture Collection (ATCC). MEFs [43] were a kind gift from C-A A. Hu (University of New Mexico, Albuquerque, NM). HeLa, and MEF cells were grown in DMEM (Gibco) supplemented with antibiotics and serum. The RGC5 cell line is a transformed rat RGC (retinal ganglion cell)-like cell line [44] was provided by B. Yue (University of Illinois at Chicago), and was initially established [45, 46]. RGC5 cells were cultured in Dulbecco’s modified Eagle’s medium, DMEM (Gibco), supplemented with serum, antibiotics and amino acids. Human corneal epithelial (HCE) cell line is an SV40-immortalized human corneal epithelial cell line previously established [47], and was kindly provided by K. Hayashi (National Eye Institute, Bethesda, MD). HCE cells were cultured in Minimum Essential Media, MEM (Gibco) supplemented with antibiotics and 10% fetal bovine serum (FBS) (Sigma).

### Viruses

HSV-1 virus strain KOS was provided by P. Spear (Northwestern University, Chicago, IL). HSV-1 virus strain McKrae was provided by H. Ghiasi (Cedars-Sinai Medical Center, Los Angeles, CA). Viruses were propagated and purified as previously described [48].

### Plasmids

pEX-GFP-hLC3WT or pEX-HcRed-hLC3WT plasmid (simply referred to as GFP-LC3, or HcRed-LC3, respectively) previously described [49] were obtained from Addgene (plasmid numbers 24987, and 24991, respectively).

### Chemicals

Saponin was purchased from Sigma. The autophagy inducer MG132 was from Selleckchem (catalog number S2619), and was used at a final concentration of 1 μM.

### Antibodies

Polyclonal antibodies against LC3 were from Novus Biologicals (Catalog number NB100-2220). Polyclonal antibodies against GAPDH were purchased from Santa Cruz (Catalog number sc-25778). Polyclonal antibodies against SQSTM1/p62 were from Santa Cruz (Catalog number sc-25575). Horseradish peroxidase-conjugated secondary (anti-rabbit) antibodies were from Jackson Immunoresearch (Catalog number 111-005-144).

### Infections

The cells were incubated with the virus in phosphate-buffered Saline (PBS) containing 0.1% glucose and 1% serum at 37°C-5% CO_2_ conditions. After 2 hrs, the virus was removed and fresh medium was added to the cells.

### Transfection

Transfections were performed using Lipofectamine2000 (Invuitrogen) according to the manufacturer’s protocols.

### Immunoblotting

Immunoblotting of proteins was performed as previously described [50]. Briefly, cells were harvested, lysed in RIPA buffer (Sigma, Catalog number R0278) containing protease-phosphatase inhibitors, and the lysates were electrophoresed through denaturing 4–12% SDS-polyacrylamide gel (Novex). Proteins were transferred onto a PVDF membrane, followed by block of non-specific binding with 5% non-fat milk in Tris-buffered saline (TBS). Membrane was then incubated with primary antibody and then with HRP-conjugated secondary antibody, followed by incubation with Femto-Sensitivity ECL (Thermo). Chemiluminescence was detected with ImageQuant LAS4000 digital image system (GE).

### Confocal fluorescence microscopy

For monitoring of LC3-GFP autophagosomal punctae, confocal microscopy (Zeiss 710 microscope, Zeiss) was used. After treatment or infection, the cells were washed, fixed in paraformaldehyde, and used in imaging. Image acquisition was performed using ZEN software (Zeiss), and image analysis was performed using MetaMorph software (Zeiss).

### Flow cytometry assay of autophagy

The assay was performed as previously described [24] and outlined in Fig 3A. Briefly, the cells were transfected with HcRed-LC3 plasmid, followed by infection or treatment with the autophagy-inducing agent (positive control). The cells were then harvested, washed briefly in the absence or presence of 0.05% saponin (for determination of total LC3, or autophagosomally localized LC3-II, respectively). The cells were then washed in FACS buffer (PBS, 1% BSA), and analyzed cytofluorimetrically on LSRFortessa cytometer (BD). Data analysis was performed using Summit software (Beckman Coulter).

### Statistical analyses

Experiments were replicated for three times. Quantification shown in figures shows mean values, and the error bars represent standard error of the mean. Student’s t-test was performed to determine statistical significance. A minimum p-value for significance was considered 0.05.
